# Development of an Effective Oral Vaccine Dissemination Strategy against Classical Swine Fever for Wild Boar in Gifu Prefecture, Japan

**DOI:** 10.1155/2023/9484441

**Published:** 2023-02-22

**Authors:** Satoshi Ito, Jaime Bosch, Cecilia Aguilar-Vega, Norikazu Isoda, Marta Martínez-Avilés, José Manuel Sánchez-Vizcaíno

**Affiliations:** ^1^VISAVET Health Surveillance Center, Complutense University of Madrid, Madrid, Spain; ^2^Department of Animal Health, Faculty of Veterinary, Complutense University of Madrid, Madrid, Spain; ^3^Laboratory of Microbiology, Department of Disease Control, Faculty of Veterinary Medicine, Hokkaido University, Sapporo, Japan; ^4^Global Station for Zoonosis Control, Global Institute for Collaborative Research and Education, Hokkaido University, Sapporo, Japan; ^5^Animal Health Research Centre (CISA-INIA/CSIC), Madrid, Spain

## Abstract

In September 2018, classical swine fever (CSF) reemerged in Japan after more than a quarter of a century. After the first notification on a pig farm, wild boars positive for CSF were found continuously in the surrounding area. Gifu was the first prefecture in Japan to disseminate oral vaccines to wild boars in March 2019, with vaccines spread to approximately 14,000 sites between 2019 and 2020. While these diligent measures seemed to have shown some effectiveness, several vaccine spray sites remained without wild boar emergence. Based on the vaccine dissemination records from these periods, this study conducted a statistical analysis to propose more effective vaccine dissemination sites. First, a generalized linear mixed model was used to identify factors correlated with wild boar emergence. Then, two spatial interpolation methods, inverse distance weighted (IDW) and Kriging, were adopted to create a probability map of wild boar emergence for the entire Gifu Prefecture. The analysis showed a positive correlation between wild boar emergence and the appearance of raccoons, raccoon dogs, and crows as well as road density and wild boar distribution index. In particular, raccoon (OR: 1.83, 95%CI: 1.25–2.68, *p* < 0.001), raccoon dog (OR: 1.81, 95%CI: 1.25–2.66, *p* < 0.001), and medium level road density (OR: 1.56, 95%CI: 1.04–2.39, *p* = 0.04) were strongly correlated with wild boar emergence. The spatial interpolation approach resulted in better prediction accuracy for the Kriging method than for IDW by the root mean square error, but both approaches identified a high wild boar appearance probability area in southeastern Gifu and a low appearance probability area in central Gifu. Here we have demonstrated a tool to effectively disperse oral vaccine to wildlife.

## 1. Introduction

Classical swine fever (CSF) is a swine disease caused by the CSF virus (CSFV), which belongs to the genus *Pestivirus* of the family *Flaviviridae*. It is considered one of the most important transboundary swine diseases, along with African swine fever (ASF) and foot-and-mouth disease, due to its high potential to cause enormous economic losses to the swine industry. The disease has been reported in many regions of the world, including Asia, Europe, Africa, and Latin America, including the Caribbean [1].

Japan successfully eliminated CSF in the past through the application of attenuated CSFV vaccines and was declared a CSF-free country in 2007. However, the first CSF outbreak in Japan in more than a quarter of a century was reported in Gifu Prefecture in September 2018[2]. The first notification was reported from a pig farm, followed by the discovery of CSF-infected wild boar carcasses in the surrounding area [3]. Subsequently, infected wild boars were found continuously in the vicinity and the number of CSF-affected farms also increased. As of the end of July 2022, 19 of the 47 prefectures had reported outbreaks in domestic pigs and 29 of the 47 prefectures reported CSF cases in wild boars [4]. The clinical symptoms of CSF-infected individuals are dependent on the virulence of the virus and biological factors of the host animals [5]. The virulence of CSFV currently prevalent in Japan is generally considered to be moderate, which means that some individuals may die as a result of CSF infection, while others may survive and play a role in further spreading the infection [6]. Despite the intensive management measures taken since the early stages of the outbreak, this complex cycle of infection has resulted in a situation where the disease is still spreading. Preventing further disease spread, the commercially available attenuated CSF bait vaccine (Pestiporc Oral, IDT Biologika GmbH, Dessau-Roßlau, Germany) [7] for wild boars was imported from Germany by the government, and the vaccination of both domestic pigs and wild boars began in October and March 2019, respectively [8]. In Gifu Prefecture, vaccine dissemination sites were selected considering the knowledge and experience of local hunters and wild boar experts, and vaccines were sprayed at a total of approximately 14,000 sites in three seasons each year from 2019 to 2020. However, wild boars positive for CSF are continuously being reported and the disease is still not under control. During each vaccination period, sensor cameras were installed at sites corresponding to approximately 10% of all vaccination sites, avoiding regional bias for the purpose of recording the emergence of wild boars. The data recorded until November 12, 2020, showed that wild boars were confirmed at only about 31% of the camera-installed sites.

The wild boar is widely distributed in Japan in evergreen broadleaf forests, deciduous broadleaf forests, secondary forests, and plains and also appears in rice paddies and agricultural land adjacent to forest areas [9, 10]. It is especially known to selectively visit broadleaf forests, abandoned paddy fields, and bamboo forests [11, 12]. Usually, wild boars get pregnant once a year in the spring or autumn and give birth to around four younglings in one birth. They are diurnal by nature but become nocturnal as a secondary habit due to human activities. Wild boars are omnivorous, digging up the ground and feeding on underground plants and animals (plant rhizomes, bamboo shoots, earthworms, etc.), acorns and other fruits, insects, frogs, snakes, and crabs. In terms of their seasonal diet, they mainly feed on bamboo shoots in spring, rice in summer, acorns in autumn, and kudzu roots in winter [13–16]. Males and females usually live separately; while young females form matrilineal groups with their mothers, males leave their mothers at 1-2 years of age and either form small groups or live alone [17]. According to the previous study, females have a home range of 67–437 ha, regardless of the various habitats, including those in other countries [18]. Wild boars are very widely distributed in Japan, and their range is presumed to still be expanding [6]. Consequently, the area reporting positive CSF cases in wild boars gradually expanded and now accounts for most parts of the main islands of Japan.

For a vaccine campaign to be effective, firstly, the vaccine itself must be effective, and secondly, wild boars should ingest the disseminated vaccine efficiently. Several publications have proven the efficacy of the vaccine in the field [19, 20]. Our current study focused on the second point; herein, statistical analysis was conducted based on available vaccine dissemination records in order to propose a more effective vaccination strategy for Gifu Prefecture as an example. First, we listed the factors that could affect wild boar emergence and clarified the relationship between each factor and wild boar emergence using a generalized linear mixed model (GLMM). Then, based on the result given by the above analysis, wild boar emergence prediction maps for the entire Gifu Prefecture were developed by spatial interpolation approaches. Thus, we proposed a tool to implement more effective vaccination.

## 2. Materials and Methods

### 2.1. Characteristics of the Study Area: Gifu Prefecture

Gifu Prefecture, the first area to report CSF in 2018, is located almost in the center of Japan. The Hida region in the northern part of Gifu Prefecture has a series of mountains over 3,000 meters above sea level, while the southern part of the prefecture is covered with plains. Because of these wide differences in elevation within the prefecture, the climate varies greatly from region to region. The forest area occupies 867,000 hectares, and 82% of the prefecture is covered by forests. A wide variety of plant communities can be found, ranging from evergreen broad-leaved forests in the warm temperate zone, deciduous broad-leaved forests in the cool temperate zone, to subarctic coniferous forests and alpine plants. This complex topography and climate have resulted in a great variety of animal and plant species living in the prefecture [21–23].

### 2.2. Data Collection

The present study was conducted using CSF oral vaccine dissemination data managed by Gifu Prefecture focusing on wild boars between March 24, 2019, and November 12, 2020. These data are not publicly available and were kindly provided by Gifu. The entire Gifu area was targeted for vaccine spraying. In 2019, oral vaccine dissemination was conducted twice in spring (March and May), summer (July and August), and winter (December 2019 and February 2020), making a total of six times. In 2020, the vaccine was disseminated once in spring (April), summer (June), and autumn (October-November). During this period, the oral vaccine was sprayed at a total of 14,131 sites in Gifu. Vaccine spray sites were determined by taking into consideration wild boar abundance, CSF epidemic situation, and distance from the farm. During each dissemination period, sensor cameras were operated to record wild boar and other animals' emergence and their vaccine feeding. These were installed at sites corresponding to approximately 10% of the total, avoiding regional bias to reflect the overall trend of the dissemination sites. These oral vaccines and installed cameras were collected 5–10 days after dissemination. The cameras were placed in standing trees in the vicinity of vaccine spray sites. During the dissemination period, each site was video recorded for 30-second and 5-second intervals, and the species and number of animals photographed were recorded. Unfortunately, the sensor camera data recorded during the vaccine dissemination period in February 2020 were not available and, therefore, not included in this study. In total, camera data including geographical *XY* coordinate, date, and animal emergence were obtained from 1227 locations during the study period.

### 2.3. Statistical Analysis Outline

The GLMM analysis was applied with a binary response (logit link) to examine the influence of predictors on the probability of wild boar emergence. The data used were collected at different times in different locations and, therefore, vaccination period and municipality of Gifu Prefecture were included in the model as temporal and spatial random factors, respectively. These were corrected for differences in wild boar behavior patterns across regions and seasonal changes as well as differences in wild boar management measures. We first selected the response variable and next listed the candidate explanatory variables. Continuous variables were categorized as necessary as described below, and single regression analysis was initially performed to screen candidate explanatory variables. Backward elimination and forward selection were then applied to construct the final multivariate model.

### 2.4. Response and Explanatory Variables

The presence or absence of wild boar emergence on sensor cameras was selected as the response variable as binary data (1 for emergence and 0 for nonemergence). The number of wild boar emergences was recorded in the data during the observation period; however, individual identification of the photographed animals was not possible and, thus, the likelihood of duplicate counts of the same individuals could not be ruled out.

Among the animal emergence and environmental data at the camera installation sites, factors that might influence the wild boar's emergence were selected as explanatory variables. The following animals were continuously observed during the study period: deer (*Cervus nippon*), bear (*Ursus thibetanus*), raccoon (*Procyon lotor*), fox (*Vulpes vulpes japonica*), raccoon dog (*Nyctereutes procyonoides viverrinus*), monkey (*Macaca fuscata*), crow (*Corvus* spp.), and badger (*Meles anakuma*). Of these animals, bear and monkey were excluded from the candidate variables due to their scarce emergences on camera and the regional bias of their distribution. The number of each animal's emergence was recorded as continuous data, but for the same reasons as for wild boars, these variables were treated as binary data.

The following environmental factors were selected as explanatory variables: altitude, slope, road density, human density, distance from water sources, wild boar distribution index, expected wild boar density, and capture pressure index. Previous studies identified elevation and slope as factors that affect wild boar habitat [24], and in Gifu, where there are large differences in elevation, these factors may be associated with their distribution. These data were downloaded as a shapefile from the National Land Numerical Information Download Service (NLNIDS) offered by the Ministry of Land, Infrastructure, Transport, and Tourism, Japan [25]. The information obtained at each camera-installed location was extracted using the Intersect tool in ArcGIS 10.8.1 [26]. While wild boars are cautious animals, once they become acclimated to the environment, they would appear even in areas inhabited by humans [27]. Human and road density may therefore influence the emergence of wild boars. These data at each site were also obtained in the same way as described above. Water is an essential element for sustaining life and, therefore, there seems to be some relationship between distance from water sources and the wild boar habitat. Here, rivers, streams, and lakes were defined as water sources, and this geographical information was downloaded from the NLNIDS. The Euclidean distance from each camera location to the nearest water source was calculated using the “Near” tool in ArcGIS 10.8.1 [28]. The wild boar distribution index was developed by multiplying the normalized difference vegetation index (NDVI) and quality of available habitat (QAH). The QAH proposed by Bosch et al. is a tool that quantifies and evaluates the optimal habitat for wild boars on seven levels (0, 0.1, 0.5, 1, 1.5, 1.75, and 2) [29]. NDVI is the index measuring the quantity, quality, and development of vegetation and, thus, frequently used as one of the predictors for the species distribution model [30, 31]. In this study, the NDVI for each sensor camera location during the study period was downloaded from the database [32]. The values of the wild boar distribution index for each location were obtained using the “Extract Multi Values to Points” tool in ArcGIS 10.8.1 [33]. In addition, environmental factors that directly affect wild boars, in this case wild boar density and capture pressure, could be relevant as predictors. While wild boars have high fertility, they also have a high mortality rate due to hunting and trapping, which results in high population fluctuations. As such, estimation of the wild boar population density in Gifu is challenging. As an alternative approach, we referred to the estimated global wild boar population density (1 km^2^ raster) data developed by Lewis [34]. As an indirect indicator of wild boar capture pressure, the density of wild boars captured in Gifu during the 200 days prior (coinciding with the period between the first wild boar case and vaccine initiation) to each vaccination was estimated using the kernel density estimation method in ArcGIS 10.8.1 [35]. As per above, the “Extract Multi Values to Points” tool in ArcGIS 10.8.1 was used to extract the values for each site.

### 2.5. Univariate Analysis

Although these candidate explanatory variables were obtained as continuous data, if a simple linear relationship is not observed between the response and explanatory variables, then categorizing the variables is recommended [36]. In this study, all continuous data except human density were categorized and the distribution of variables was equally divided into four so that each category had approximately equal sample sizes. However, if there is a large imbalance in the data, it is not possible to divide the data into equal parts. For the variables of expected wild boar density and the capture pressure index, classification by Jenks natural breaks [37, 38] placed the data into four categories.

A single regression analysis was performed to examine the relationship between each explanatory and response variable. Variables with *p* values greater than 0.15 were excluded at this step. In the case of categorical data, the relation to the response variable may not be clear depending on the values taken by the variables. In such cases, a model with the explanatory variable of interest and a null model without an explanatory variable were compared by likelihood ratio tests. The null hypothesis is “of the two models compared, the more complex model has the same or less fit to the data than the less complex model.” If the *p* value was very small (less than *p* *=* 0.05), the null hypothesis was rejected, and the variable of interest was selected as a candidate for inclusion in the multivariate model. This step was performed with the “lrtest” function in the “lmtest” package in the R programming environment [39].

### 2.6. Multivariate Model

The GLMM analysis was performed with the “glmer” function in the “lme4” package in an R programming environment [40]. The explanatory variables obtained from the univariate analysis were used as fixed effects, while the municipality of Gifu Prefecture and vaccination period were selected as random effects. First, an analysis for the variance inflation factor (VIF) was performed with the “vif” function in the “car” package in R [41] to avoid multicollinearity among the explanatory variables. Here, variables for which VIF >5 were used as explanatory variables in the model. A backward elimination and forward selection procedure was then performed and, at each step, the best model was selected by running the “anova” function in the “lme4” package (criterion: *p* value <0.05).

### 2.7. Evaluation of the Model

The Hosmer–Lemeshow test was used to determine the goodness of fit of the model. Fundamentally, this is a chi-square goodness-of-fit test for grouped data, typically where the data are partitioned into 10 equal subgroups. As a complementary approach, a receiver operating characteristic (ROC) curve was drawn to evaluate the productivity of the model. ROC is a prediction curve that plots the ratio of the true positive rate on the vertical axis and false positive rate on the horizontal axis. AUC (area under the curve) is the area under the ROC curve, and a larger AUC area generally means better prediction performance. An AUC of 1 (a model with perfect discriminability) is the most optimal, and a value of 0.5 implies no better than a random model [42].

### 2.8. Spatial Autocorrelation Analysis

To understand spatial autocorrelations in the resulting values of the response variables at each location, Global Moran's I tool was performed in ArcGIS 10.8.1 [43, 44]. As a preliminary step, obtained values of wild boar emergence probability for each site were linked to the geographic XY coordinates of the sensor cameras and projected in ArcGIS.

### 2.9. Predicted Wild Boar Emergence Surface in Gifu by the Inverse Distance Weighted (IDW) Approach

Inverse distance weighted (IDW) interpolation is a spatial interpolation method called deterministic interpolation as the surrounding measurements determine the smoothness of the output surface and directly affect the results [45]. The hypothesis regarding spatial relationships here is that values closer to one value to be interpolated are more relevant than values farther away. The estimated value *z* at location *X* is a weighted average of nearby observations:

(1) $$\begin{array}{c} \hat{Z}\left(X\right)=\frac{\sum_{i}^{n}w_{i}z_{i}}{\sum_{i}^{n}w_{i}}\text{,}\\ w_{i}={\left|X-X_{i}\right|}^{-\beta }\text{,} \end{array}$$

where *β* ≥ 0 and |·| corresponds to the Euclidean distance. The inverse distance power, *β*, determines the degree to which the nearer points are preferred over more distant points [46]. The analysis was performed for the entire Gifu region, specifying the wild boar emergence probability at each location as the interpolated value. All calculations were performed by the “idw” function of the “gstat” package in R [47]. Surface mapping was performed in ArcGIS 10.8.1.

### 2.10. Predicted Wild Boar Emergence Surface in Gifu by the Kriging Approach

Kriging is a multistep approach structured into a geostatistical method accounting for spatial autocorrelation [48–50], different from deterministic interpolation methods such as IDW (45, 46), and has been applied in a wide range of fields [51–53].

For a point of *S*_*i*_(*i*=1,…, *n*) in the continuous space, let *X*_*k*_(*S*_*i*_)(*k*=1,…, *n*) be the variables related to *S*_*i*_ and let *h*_*ij*_=‖*s*_*i*_ − *s*_*j*_‖ be the distance between *ij*. Suppose that the stationary process *Z*(*S*_*i*_) with respect to *S*_*i*_ is expressed as

(2) $$\begin{array}{c} Z\left(S_{i}\right)=\overset{m}{\underset{k=1}{\sum }}X_{k}\left(S_{i}\right)\beta_{k}+\varepsilon \left(S_{i}\right)\text{,}\\ \varepsilon \left(S_{i}\right)∼N\left(0,\sigma_{i}^{2}\right)\text{.} \end{array}$$

Then, the semivariance (*h*_*ij*_) is calculated as the difference squared between the values of the paired locations allowing to express the autocorrelation structure of the variables between the locations:

(3) $$\begin{array}{c} \gamma_{i}\left(h_{ij}\right)=\frac{1}{2}E{\left[Z\left(S_{i}\right)-Z\left(S_{i}+h_{ij}\right)\right]}^{2}\text{.} \end{array}$$

Then, the semivariance $\hat{\gamma }\left(\tilde{h}\right)$ of the entire point can be expressed by the number of combinations *N*_*h*_ of points *Z*(*S*_*i*_) and *Z*(*S*_*i*_+*h*_*ij*_), which are distances, as follows [54]:

(4) $$\begin{array}{c} \hat{\gamma }\left(\tilde{h}\right)=\frac{1}{2N_{h}}\overset{N_{h}}{\underset{i=1}{\sum }}\gamma_{i}\left(h\right)\text{.} \end{array}$$

As with the IDW method, the probability of wild boar emergence at each site was specified as the interpolated value, and the analysis was conducted for the entire Gifu region. First, a variogram cloud was created using the variogram function of the “gstat” package in R to evaluate pairs of sample values. Next, an experimental variogram was created using the “variogram” function of the same package. At a certain distance (range), autocorrelations become independent, meaning that when their variation levels off (sill), there is no longer any spatial autocorrelation between the proximities of the data points. The experimental variogram was used to fit a theoretical variogram model to estimate several parameters (sill, range, and nugget) representing the range of autocorrelation and variance. The nugget is the *y* intercept of the variogram and is thought to be due to measurement errors or sources of spatial variation at distances shorter than the sampling interval. In the step of creating variogram models using the “vgm” function, commonly selected models “Exp,” “Gau,” and “Sph” were specified and the best model was picked using the “fit.variogram” function. Based on the obtained model and the default values of the parameters (sill, range, and nugget), both the experimental variogram and the model were plotted and overlaid to evaluate the initial model. The model was then fitted using one of the fit criteria, and ordinary Kriging was performed with the krige function in the same package. Surface map creation was performed in ArcGIS 10.8.1.

### 2.11. Cross-Validation

We compared and evaluated the prediction accuracy of IDW and Kriging methods using leave-one-out cross-validation, a process that first takes one point from the calibration data, second, predicts that point, and third, repeats the same process for all points [55]. The final results were obtained as tables containing the observed and predicted values for all points. The validation was performed for each method using the “gstat.cv” function from the “gstat” package. The predicted and observed columns of the resulting table were used to calculate the root mean square error (RMSE) [51].

## 3. Results

### 3.1. Univariate Analysis

Single regression analysis showed that variables of raccoon, raccoon dog, crow, altitude, slope, road density, capture pressure index, expected wild boar density, and wild boar distribution index were significantly correlated with the response variable of wild boar emergence at the sensor camera locations in Gifu (*p* value less than 0.15) (Table 1). The environmental factors mentioned above were subsequently performed with a likelihood ratio test against the null model (*p* value less than 0.05). In this step, the variable altitude was excluded from the candidate explanatory variables (*p* *=* 0.293). The remaining variables were selected as candidates for inclusion in the multivariate model.

### 3.2. Multivariate Analysis

The VIF analysis was performed, and all factors had a VIF of less than 1.1, and thus no explanatory variables were excluded in this step. In the backward elimination and forward selection procedure, the capture pressure index variable was first excluded (*p* *=* 0.254), the slope variable was excluded secondly (*p* *=* 0.153), and the expected wild boar density variable was excluded thirdly (*p* *=* 0.072). At this point, all variables met the criterion of *p* value less than 0.05 (Table 2.). Finally, a GLMM was constructed with variables of raccoon, raccoon dog, crow, road density, and wild boar distribution index as fixed effects and incorporating vaccination period and municipality as random effects. The results of the model performance showed that the appearance of raccoon (OR: 1.83, 95%CI: 1.25–2.68, *p* < 0.001), raccoon dog (OR: 1.81, 95%CI: 1.25–2.66, *p* < 0.001), and crow (OR: 1.36, 95%CI: 1.02–1.81, *p* = 0.04) was positively correlated with the emergence of wild boar. In the road density variable, we found that higher road density was positively correlated with wild boar emergence. Among the variables of road density, medium road density (586–2103 m/km^2^) had the highest correlation with wild boar emergence (OR: 1.83, 95%CI: 1.25–2.70, *p* = 0.03), followed by medium-high (OR: 1.56, 95%CI: 1.04–2.39, *p* = 0.04) and high (OR: 1.57, 95%CI: 1.04–2.38, *p* = 0.002) road density. For the wild boar distribution index, there was a significant positive correlation for wild boar emergence in areas with medium level (OR: 1.46, 95%CI: 1.01–2.10, *p* = 0.04). On the other hand, areas with medium-high and high levels were not statistically significant (*p* > 0.05).

### 3.3. Evaluation of the Model

**Figure 1:**
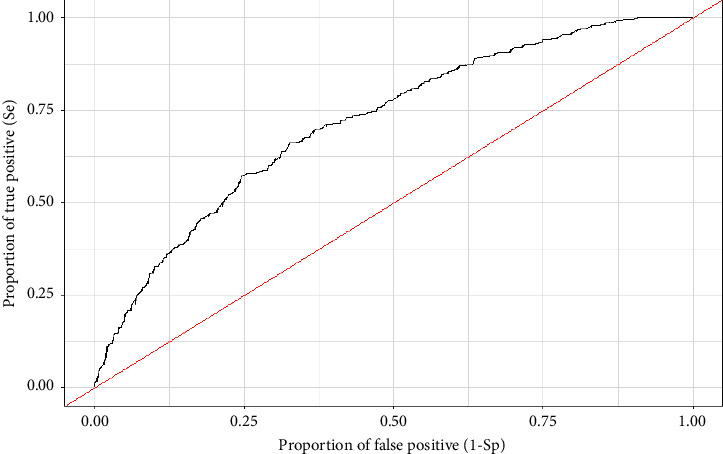
ROC curves (receiver operating characteristic curve) for generalized linear mixed models were plotted with true positive ratio (Se) against false positive ratio (1-Sp) where Se is on the *y*-axis and 1-Sp is on the *x*-axis.

The null hypothesis of the Hosmer–Lemeshow test is that “the model fits the data.” If the null hypothesis is rejected by a statistical test (i.e., the *p* value is less than 0.05), the model does not fit the data. The results of the present analysis gave a *p* value = 0.204, thus indicating the validity of our model. The ROC curve was drawn based on the multivariate model obtained, and the AUC was calculated as 0.717 (Figure 1).

### 3.4. Spatial Autocorrelation Analysis

The values of response variables obtained from the multivariate analysis were associated with the geographic coordinates of each sensor camera location. These locations were first plotted and projected in ArcGIS 10.8.1. Spatial autocorrelation among the response variables was analyzed using Global Moran's I tool, resulting in Moran's I index = 0.379, *z*-score = 10.82, and *p* < 0.001. Thus, spatial autocorrelation was among the response variables.

### 3.5. Predicted Wild Boar Emergence Surface in Gifu by the IDW and Kriging Approaches

**Figure 2:**
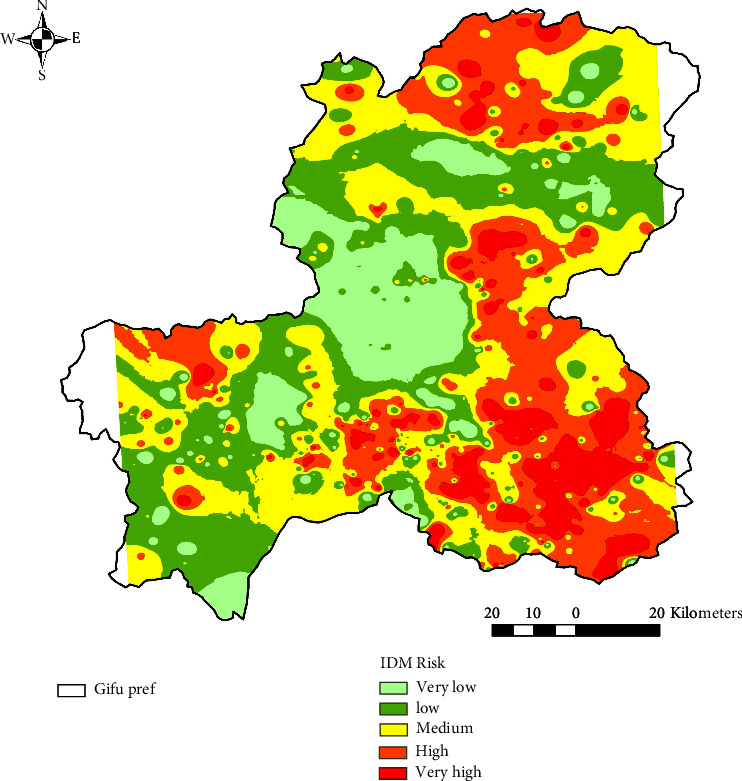
The predictive surface of wild boar emergence probability in Gifu Prefecture by the IDW (inverse distance weighted) approach was depicted on ArcGIS 10.8.1. Each colored area indicates the probability of wild boar emergence from the lowest (light green) to the highest (red): very low (0.133–0.227), low (0.227–0.299), medium (0.299–0.362), high (0.362–0.440), and very high (0.440–0.792).

The wild boar emergence probability map for Gifu was interpolated by the IDW approach and was depicted on ArcGIS 10.8.1. The relative probability score of wild boar emergence from very low to very high was assigned based on Jenks natural breaks. Each colored area indicates the probability of wild boar emergence: very low (0.133–0.227), low (0.227–0.299), medium (0.299–0.362), high (0.362–0.440), and very high (0.440–0.792) (Figure 2). As a result, areas with relatively high wild boar emergence probability (very high-high) were observed in the southeastern part of Gifu. These high probability areas were also found scattered in the southern, northern, and western parts of Gifu Prefecture. On the other hand, large areas with relatively low wild boar emergence probability (very low-low) were observed in the central part of Gifu.

**Figure 3:**
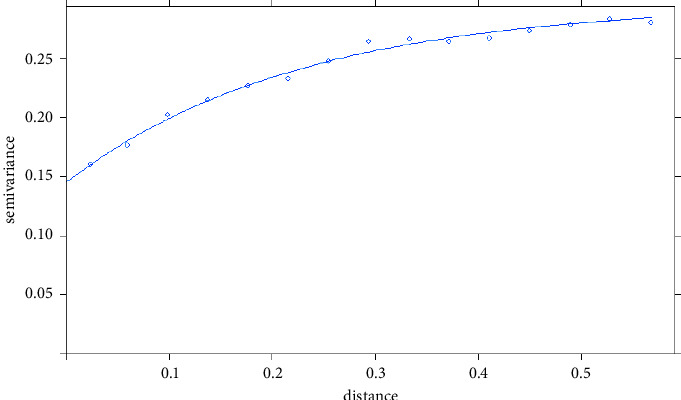
Fitted theoretical variogram with exponential model of the ordinary Kriging approach.

**Figure 4:**
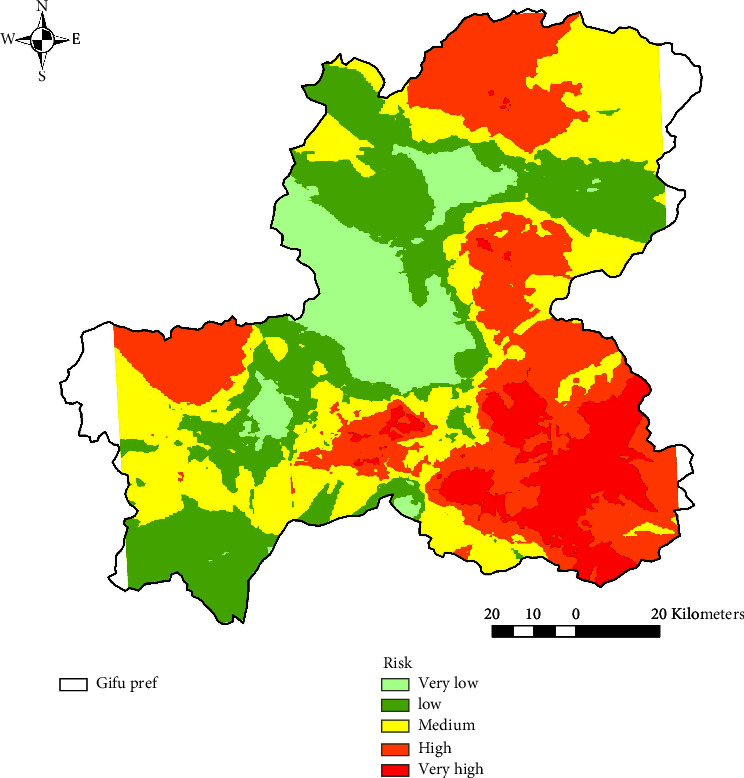
The predictive surface of wild boar emergence probability in Gifu by the Kriging approach was depicted on ArcGIS 10.8.1. Each colored area indicates the probability of wild boar emergence from the lowest (light green) to the highest (red): very low (0.133–0.227), low (0.227–0.299), medium (0.299–0.362), high (0.362–0.440), and very high (0.440–0.792).

In the Kriging approach, after approximating the experiential semivariogram with a model, “Exp” was chosen as the best model, with values of 0.144, 0.296, and 0.226 for nugget, sill, and range, respectively (Figure 3). By using these values, a prediction surface of wild boar emergence probability for Gifu Prefecture was depicted on ArcGIS 10.8.1 (Figure 4). The same classification method and criteria as the IDW method were applied to assign five probability scores (very low–very high). As a result, areas with relatively high wild boar emergence probability (very high-high) were observed in the southeastern part of Gifu. High probability areas were scattered near the northern, eastern, southern, and southwestern prefectural borders. As with the IDW method, large areas with relatively low wild boar emergence probability (very low-low) were observed in the central part of Gifu.

Finally, we evaluated the prediction accuracy of the IDW and Kriging approach with leave-one-out cross-validation. The results showed that RMSEs for IDW and Kriging were 0.141 and 0.129, respectively, indicating that Kriging had a better prediction accuracy than IDW in this analysis.

## 4. Discussion

Since CSF reemerged in Japan, research on the disease has been initiated from various perspectives. Analysis of the pathogenicity of the virus [56], clarification of the mechanism of transmission [3, 5], and studies of the transmission speed and basic reproduction rate [8, 57, 58] have all been significant studies for disease control. Many of these studies also mentioned the effectiveness of the measures implemented. On the other hand, the knowledge concerning vaccination strategies is very limited. The selection of vaccination sites can be a very important issue if maximum efficiency is to be achieved in controlling long-lasting CSF outbreaks.

GLMM analysis revealed that the variables of raccoon dog, raccoon, crow, road density, and wild boar distribution index significantly contributed to wild boar emergence at each site. In particular, the presence of raccoon dogs and raccoons increased the likelihood of wild boar emergence by approximately 1.8-fold each. This is probably due to their commonality in food resources and habitat area. The medium level of road density showed the highest correlation among road density variables, which seems reasonable considering the behavioral patterns of wild boars. These areas are presumably part of the area connecting urban and forest areas. It is well known that wild boars, while using forested areas as their habitat, also roam around human settlements seeking food resources [59, 60]. Accordingly, areas belonging to these medium level road densities could be corridors along which wild boars frequently move [59, 60]. As one of the explanatory variables, we selected the wild boar distribution index, which indicates areas suitable as wild boar habitats during the study period. A positive correlation was found between areas belonging to the medium level of the wild boar distribution index and the response variable. The high level of the wild boar distribution index indicates an abundance of food resources and residential areas suitable for wild boar, while areas with medium levels are assumed to have insufficient food resources or habitation areas. This may have resulted in the movement of wild boars and an increase in their emergence at the sensor camera locations. Notably, the animal variables (raccoon dog and raccoon) showed a higher correlation with the response variables than the wild boar distribution index variable. Given that QAH is a more generalized tool for the entire Eurasian continent, the habitat distribution of these animals may indirectly indicate more regional food resources or habitat area preferences for wild boars. As evidenced, Japanese wild boars are known to prefer bamboo shoots in the spring and frequently appear in rice paddies [15], which seems to be a unique behavioral pattern, different from that in Europe. These results suggest that there is no universal standard to identify the best vaccine spray areas, and therefore designing a dissemination strategy that fully takes into account regional characteristics is important. The expected wild boar density and capture pressure index variables, which are indicators of wild boar population density and capture pressure, were excluded in the final model selection step. Wild boar densities are highly changeable [61], and capture pressure is also greatly influenced by the epidemic situation. As such, obtaining accurate information about these factors is challenging. The current analysis concluded that wild boar emergence is greatly influenced by their abundance rather than population density; note, however, that these results may be influenced by the quantity and quality of the data. The results of the Hosmer–Lemeshow test (*p* *=* 0.204) and AUC (0.717) prove the validity of this model, and the prediction accuracy is fair [62], but further investigation to improve the goodness of fit would be needed. Specifically, the ecological knowledge about wild boars is very limited, and concern remains that our estimation of the population distribution of wild boars is simplistic. To cite an example, changes in the behavioral patterns of wild boars associated with seasonal changes and the breeding season, which were incorporated as a random effect in this study, are expected to affect their range and feeding behavior. There is a potential selection bias because these vaccine dissemination sites inevitably reflect accessibility from human settlements as well as wild boar habitats. Studies addressing these issues are crucial to reflect the real situation.

Analysis of spatial autocorrelation by Moran's I tool revealed that the values of response variables were clustered at locations where sensor cameras were installed. Consequently, we developed a wild boar emergence prediction surface in Gifu Prefecture using two spatial interpolation methods, the IDW and the Kriging methods. High wild boar emergence risk areas in southeastern Gifu Prefecture and low-risk areas in central Gifu Prefecture were identified by both methods. At the same time, the IDW method further indicated that high-risk areas are scattered throughout the prefecture. In the Kriging method, the values of interpolated points are determined by their spatial autocorrelation with each other, while in the IDW method, the influence is determined by multiplying the inverse of the distance by a power. These algorithmic differences probably influenced the results. The results of the RMSE cross-validation concluded that the prediction accuracy of Kriging was higher in this study. According to [63], the accuracy of both IDW and Kriging is very similar; while IDW has the advantage of being intuitive and efficient, Kriging is preferable for more informative data and provides highly reliable interpolation [63]. In fact, the accuracy of the method appears to vary depending on the quantity and quality of the dataset [64, 65].

The experience in Europe cannot be directly applied to Japan as described in other papers [8]. Germany, which has successfully controlled CSF in wild boar, has a relatively flat terrain compared with Japan. In addition, Germany has a background of game hunting culture, and supplemental feeding on wild boars is a common practice. The areas where wild boars gather are well known, so effective bait vaccine dissemination can be expected. On the other hand, Japan's terrain is full of steep, sheer mountains; many wild boars presumably inhabit areas that are inaccessible to humans. Additionally, the practice of feeding wild boars is not very common. For these reasons, wild boars in Japan would be more cautious of humans. Therefore, even if oral vaccines are sprayed, they may not approach areas where human traces are present. Vaccine spray should be carefully conducted in consideration of these regional characteristics. Furthermore, we should keep in mind that the emergence of wild boars and their vaccine-feeding rate are completely separate issues. The presence of raccoon dogs, raccoons, and crows positively correlated with the emergence of wild boars, but these animals can be competitors when focusing on the vaccine-feeding rate by wild boars. Therefore, apart from identifying optimal vaccine spray sites, countermeasures to prevent vaccine ingestion by these animals need to be designed. In Gifu, stones have been placed at vaccine dissemination sites as a measure against small animals. The effectiveness of such measures should also be examined. Oral vaccination has proven to be effective in maintaining population immunity and CSF control and is considered the only method for CSF eradication in large forested areas [8, 66–68]. While several publications have described insights into wildlife vaccination in Europe, these insights are very limited in Asian countries, where both geographical conditions and ecosystems are considerably different from those in Europe [67–69].

From an economic standpoint, the primary concern would be the economic loss to the swine industry; however, keep in mind that this is closely related to the spread of CSF in wild boars. The major problem facing Japan today is that CSF expansion among wild boar populations greatly affects the epidemic status of CSF in pig farms [57, 70]; hence, spreading the disease in the field remains a threat to the swine industry. The final objective of the Japanese Government is to be certified as a CSF-free country under WOAH regulations [71]. Control of CSF in the field based on effective vaccine dissemination strategies is the first key toward disease eradication. Our study is an important first step in this direction.

The present study focused on wild boar vaccination against CSF, but the proposed tool may also be useful for controlling other diseases. Currently, ASF outbreaks continue to expand in Europe and Asia. Our previous studies demonstrated that it is quite possible that ASF outbreaks could occur in Japan [72, 73]. When an ASF vaccine for wild boar is commercialized and used in the field, our established tools will have greater importance.

## Figures and Tables

**Table 1:** The results of single regression analysis.

Variables	Variable type 1	Variable type 2	Unit	Variable (threshold)	*N* = 1227 (%)	Coefficients (*β*)	Standard error (SE)	*p*value	Data source
Wild boar	Response	Binominal	Presence: yes or no	Yes	398 (32.4%)	—	—	—	Camera data
				No	829 (67.6%)	—	—	—	
Deer	Explanatory	Binominal	Presence: yes or no	Yes	256 (20.9%)	0.133	0.148	0.371	Camera data
				No	971 (70.1%)	—	—	—	
Raccoon dog	Explanatory	Binominal	Presence: yes or no	Yes	986 (80.4%)	0.734	0.172	**<0.001**	Camera data
				No	241 (19.6%)	—	—	—	
Raccoon	Explanatory	Binominal	Presence: yes or no	Yes	165 (13.4%)	0.703	0.170	**<0.001**	Camera data
				No	1062 (86.6%)	—	—	—	
Fox	Explanatory	Binominal	Presence: yes or no	Yes	683 (55.7%)	0.143	0.123	0.246	Camera data
				No	544 (44.3%)	—	—	—	
Crow	Explanatory	Binominal	Presence: yes or no	Yes	392 (31.9%)	0.416	0.128	**<0.001**	Camera data
				No	835 (68.1%)	—	—	—	
Badger	Explanatory	Binominal	Presence: yes or no	Yes	188 (15.3%)	5.4 × 10^−4^	0.169	0.997	Camera data
				No	1039 (84.7%)	—	—	—	
Altitude	Explanatory	Categorical	Meter	Low: 0–25% (993.5)	308 (25.1%)	—	—	—	[25]
				Medium: 25–50% (1912.8)	308 (25.1%)	0.329	0.173	**0.058**	
				Medium-high: 50–75% (2166.3)	304 (24.8%)	0.218	0.175	0.213	
				High: 75–100% (2529.2)	307 (25.0%)	0.174	0.176	0.322	
Slope	Explanatory	Categorical	%	Low: 0–25% (5.7)	309 (25.2%)	—	—	—	[25]
				Medium: 25–50% (12.7)	306 (24.9%)	−0.166	0.173	0.339	
				Medium-high: 50–75% (22.7)	305 (24.9%)	−0.272	0.176	**0.121**	
				High: 75–100% (39.6)	307 (25.0%)	0.194	0.168	0.249	
Road density	Explanatory	Categorical	m/km^2^	Low: 0–25% (586)	307 (25.0%)	—	—	—	[25]
				Medium: 25–50% (2103)	307 (25.0%)	0.68	0.178	**<0.001**	
				Medium-high: 50–75% (4399)	307 (25.0%)	0.568	0.179	**<0.002**	
				High: 75–100% (9011)	306 (24.9%)	0.427	0.181	**0.019**	
Distance to water source	Explanatory	Categorical	Meter	Short: 0–25% (88.2)	310 (25.3%)	—	—	—	[25]
				Middle: 25–50% (211.6)	306 (24.9%)	−0.024	0.170	0.888	
				Middle-long: 50–75% (382.3)	305 (24.9%)	−0.250	0.175	0.153	
				Long: 75–100% (1491.6)	306 (24.9%)	0.005	0.03	0.976	
Capture pressure index	Explanatory	Categorical	Head/km^2^	Low density (<0.252)	942 (76.8%)	—	—	—	Gifu-provided data
				Medium density: (0.252–0.805)	235 (19.2%)	0.239	0.153	**0.120**	
				High density: (0.805–1.82)	41 (3.3%)	1.070	0.323	**<0.001**	
				Very high density: (>1.82)	9 (0.7%)	0.602	0.675	0.372	
Expected wild boar density	Explanatory	Categorical	Head/km^2^	Low density: (<5)	167 (13.6%)	—	—	—	[34]
				Medium density: (5-6)	400 (32.6%)	0.094	0.200	0.640	
				Medium-high density: (6-7)	306 (24.9%)	−0.349	0.164	**0.033**	
				High density: (>7)	354 (28.9%)	−0.060	0.164	0.715	
Human density	Explanatory	Continuous	Person/km^2^	(min, median, max)	0, 2, 892	6.7 × 10^−4^	7.7 × 10^−4^	0.383	[25]
Wild boar distribution index	Explanatory	Categorical		Low: 0–25% (0.571)	307 (25.0%)	—	—	—	[29, 32]
				Medium: 25–50% (0.612)	307 (25.0%)	0.359	0.170	**0.035**	
				Medium-high: 50–75% (0.646)	306 (24.9%)	−0.073	0.176	0.680	
				High: 75–100% (1)	307 (25.0%)	−0.031	0.175	0.861	

Variables with *p* values less than 0.15 (in bold) were advanced to the next step.

**Table 2:** The results of multiregression analysis.

Variables	Variable type	Unit	Variable	*N* = 1227 (%)	*β*	SE	Odds ratio (95% CI)	*p*value	Data source
Raccoon dog	Binominal	Presence: yes or no	Yes	986 (80.4%)	0.594	0.191	1.81 (1.25–2.66)	**<0.001**	Camera data
			No	241 (19.6%)	—	—		—	
Raccoon	Binominal	Presence: yes or no	Yes	165 (13.4%)	0.603	0.195	1.83 (1.25–2.68)	**<0.001**	Camera data
			No	1062 (86.6%)	—	—		—	
Crow	Binominal	Presence: yes or no	Yes	392 (31.9%)	0.306	0.146	1.36 (1.02–1.80)	**0.036**	Camera data
			No	835 (68.1%)	—	—		—	
Road density	Categorical	m/km^2^	Low: 0–25%	307 (25.0%)	—	—		—	[25]
			Medium: 25–50%	307 (25.0%)	0.605	0.196	1.83 (1.25–2.70)	**0.002**	
			Medium-high: 50–75%	307 (25.0%)	0.446	0.206	1.56 (1.04–2.39)	**0.031**	
			High: 75–100%	306 (24.9%)	0.449	0.211	1.57 (1.04–2.38)	**0.033**	
Wild boar distribution index	Categorical		Low: 0–25%	307 (25.0%)	—	—		—	[29, 32]
			Medium: 25–50%	307 (25.0%)	0.377	0.185	1.46 (1.01–2.10)	**0.042**	
			Medium-high: 50–75%	306 (24.9%)	−0.174	0.194	0.84 (0.57–1.23)	0.370	
			High: 75–100%	307 (25.0%)	−0.180	0.194	0.84 (0.57–1.22)	0.353	

Variables with *p* values less than 0.05 are shown in bold.

## Data Availability

The data applied in this study are not publicly available and were kindly provided by Gifu Prefecture, Japan.
